# CONFIRMS: A Toolkit for Scalable, Black Box Connectome Assessment and Investigation

**DOI:** 10.1109/EMBC46164.2021.9630109

**Published:** 2021-11

**Authors:** Caitlyn Bishop, Jordan Matelsky, Miller Wilt, Joseph Downs, Patricia Rivlin, Stephen Plaza, Brock Wester, William Gray-Roncal

**Affiliations:** 1 Johns Hopkins University Applied Physics Laboratory, Laurel, MD 20723, USA; 2 Janelia Research Campus, Ashburn, VA 20147, USA

## Abstract

The nanoscale connectomics community has recently generated automated and semi-automated “wiring diagrams” of brain subregions from terabytes and petabytes of dense 3D neuroimagery. This process involves many challenging and imperfect technical steps, including dense 3D image segmentation, anisotropic nonrigid image alignment and coregistration, and pixel classification of each neuron and their individual synaptic connections. As data volumes continue to grow in size, and connectome generation becomes increasingly commonplace, it is important that the scientific community is able to rapidly assess the quality and accuracy of a connectome product to promote dataset analysis and reuse. In this work, we share our scalable toolkit for assessing the quality of a connectome reconstruction via targeted inquiry and large-scale graph analysis, and to provide insights into how such connectome proofreading processes may be improved and optimized in the future. We illustrate the applications and ecosystem on a recent reference dataset.

## Clinical relevance—

Large-scale electron microscopy (EM) data offers a novel opportunity to characterize etiologies and neurological diseases and conditions at an unprecedented scale. EM is useful for low-level analyses such as biopsies; this increased scale offers new possibilities for research into areas such as neural networks if certain bottlenecks and problems are overcome.

## I. Introduction

One of the primary challenges of large-scale connectomics (i.e., mapping the brain from nanoscale neuroimaging data) is 3D volumetric network reconstruction. Misclassifications such as merges and splits can have profound consequences for the validity of the resulting connectome. Despite many recent advances in 3D image segmentation [1], [2], [3], human expert annotation is still a requirement in order to guarantee large-scale, high-confidence neurite traces [4], [2], [5], [6]. This human intervention is a significant bottleneck: recent projects, such as the IARPA MICrONS visual cortex volume and the Janelia Hemibrain partial fly brain required many staff years of manual segmentation labor in order to achieve high-quality segmentations [5], [6], [7], [8]. The community has developed various specialized measures [1], [9] to evaluate the validity of a putative connectome; proofreading remains essential to correct many types of errors.

As connectomics datasets continue to grow in size, it will become increasingly important that quality assessment technologies scale rapidly to accommodate the petabyte-scale volumetric datasets being produced by the community. To meet this need, we propose a solution that marries supervised (human-in-the-loop) and automated connectome assessment. Here, we present our tools and share important lessons learned for future connectomics evaluators. We note that as graph reconstructions become larger, proofreading as understood today may be infeasible to do exhaustively. Our forced-choice approaches allow us to do focused proofreading [10], [11], and also assess the completeness and quality of the data, toward results that may be more rapidly characterized and used by the computational neuroscience, clinical, and machine learning communities.

### A. Background

The field of neuroscience has long sought a complete “wiring diagram” of the brain. One representation that has grown in popularity is a graph representation, where neurons are represented by nodes, and synapses are represented as directed edges [12], [13]. A dense reconstruction of even a modestly sized 3D volume of neural tissue comprises an enormous amount of manual labor if segmented exclusively by humans [14], [15], and so connectomics researchers generally leverage automated (often machine-learning based) tools to perform many of the key steps of connectome generation [1], [7], [16], [17].

Modern connectomics datasets often reach the multi-terabyte scale, and commonly include hundreds of teravoxels of data [18], [19]. It is therefore unlikely that the entire dataset will ever be examined by human eyes at pixel resolution. These datasets empower neuroscientists to focus on key research questions at a previously impossible scale, but introduce new challenges. For example, researchers must consider how to frame machine learning tasks with training datasets far smaller (and possibly not representative) of the larger inference volume. Indeed, in many cases, neuroscience researchers have encountered completely novel anatomical phenomena in part thanks to automated connectome generation tools [6], [7].

**Fig. 1.**
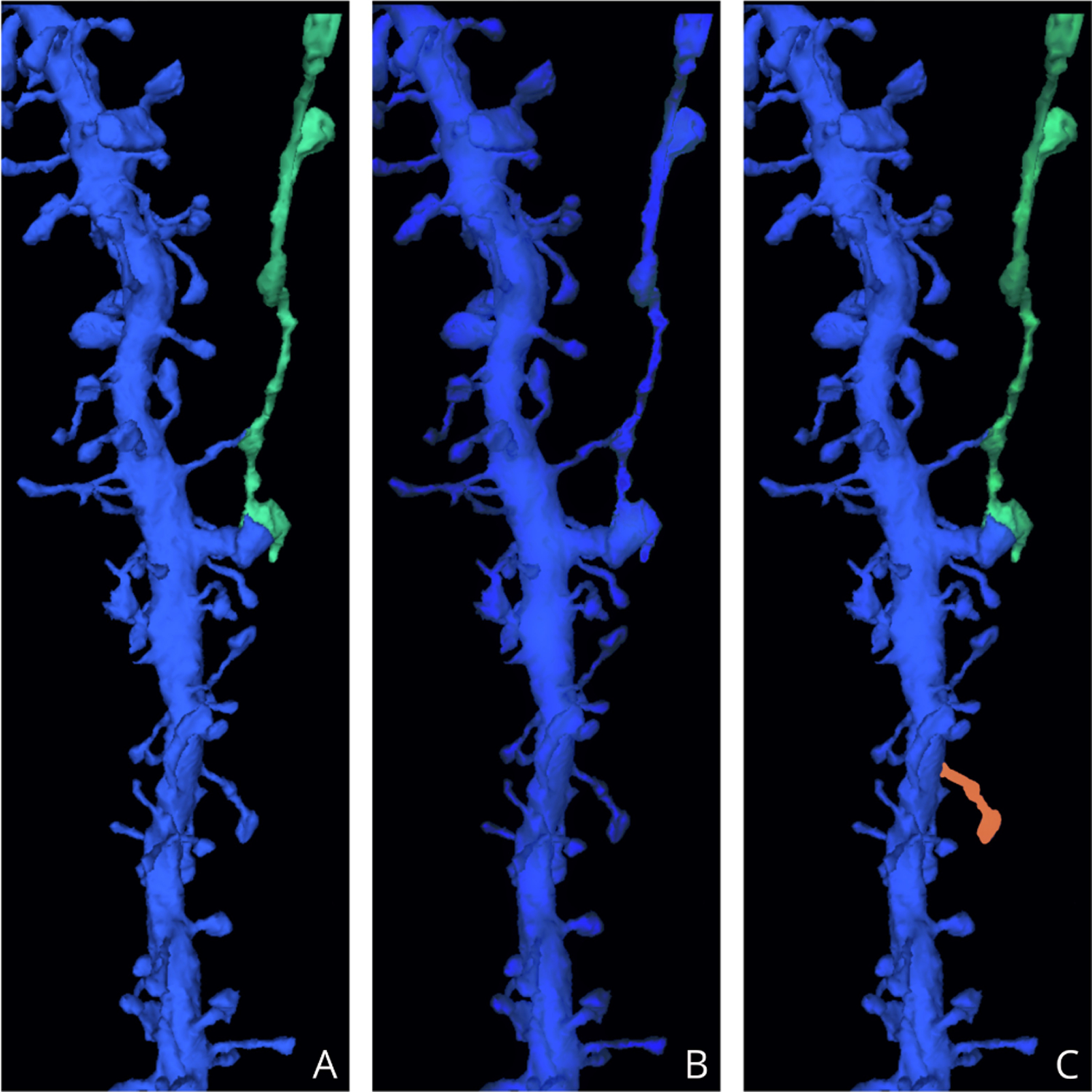
Synthetic examples illustrating common segmentation errors resulting from automated segmentation methods [22], [4] (a: ground truth, b: false merge, c: false split)

In recent years, 3D image segmentation has emerged as the primary machine learning challenge and bottleneck for electron microscopy connectomics [1], [20], [21]. Segmentation failures are often classified into one of two failure types: merge errors, in which two or more different cells are incorrectly assigned the same segmentation ID, or split errors, in which different regions of a single cell are assigned distinct segmentation IDs (Figure 1). Identifying these errors after segmentation is still a difficult problem, and many recent efforts [4], [5], [6] have relied upon manual human proofreading to correct segmentation mistakes.

Some metrics, such as Expected Run Length (ERL) [1] have been developed to quantify the accuracy of a segmentation. ERL is the expected distance one can traverse through Euclidean space before encountering a merge or split error (i.e., before entering a new neuron assigned the same segmentation ID, or before encountering a segmentation ID change within a single neuron). Other workflows operate on a post-processing step, which enables them to use higher-level semantic heuristics. For example, Neural Reconstruction Integrity (NRI) is a metric that produces an expected connectome accuracy measure while penalizing merge and split errors specifically [9]. Additional conventional metrics, such as pixel-wise f1 scores, are often employed to supplement these findings [16].

## II. Tool Overview

We developed generalized tools and API endpoints that are agnostic to the underlying semantic meaning of the data. This will enable these tools to be reused by the community in future projects and adopted by new communities for keypoint and graph annotation and machine learning dataset generation. For example, we used the terminology of keypoints rather than “synapses,” and graph or network terminology instead of “neuron” or “skeleton.” At several points during the maturation of this software suite, we were able to repurpose tools thanks to this generalized design (for example, reusing the keypoint applications, which were originally designed for synapse detection, in order to train a soma detection algorithm at much lower spatial resolution). This generalized software architecture involved server-side patterns as well as client-side patterns.

### A. Queue & API Design

We developed a specialized task queue and results storage system for our proofreading workflows, leveraging industry standard schemaless datastore MongoDB. Our initial designs were written to accommodate real-time feedback over websockets, but we discovered that few relevant use-cases necessitated this type of feedback, and indeed too much visual information served only to distract annotators. The software is currently designed using TypeScript and MongoDB, and is served by an Express application server. To accommodate bursts of high-throughput user proofreading activity, all server-side tools are packaged in Docker images, and multiple endpoints may be provisioned in order to serve higher-than-average demand. We also include database backup and restoration utilities to enable administrators to take snapshots of the database and back up results to external storage.

Our neuroimaging volumes are served from BossDB infrastructure [18] via authenticated REST API. Authentication is a conventional OAuth system that may be linked to an external authentication engine and easily adapted to other backends or tasks. Authenticated users also benefit from differential access to the database: Proofreader users can only write new data and read tasks relevant to their user accounts. Administrative users, or task authors, can read and write data for multiple users. More fine-grained controls are available to support additional future use-cases.

### B. Database Design

Our database schema was designed to be maximally flexible and entirely agnostic to underlying data types or modalities. We developed a generalized data type schema composed of Volumes, Graphs, Nodes, Questions, and Decisions. All data types include the option to store arbitrary metadata in the form of key-value maps, as well as the name of the user that created the object (“author”). Annotatable data types (graphs, nodes, and decisions) also include the user assigned to annotate that item (“assignee”). A complete schema is available in the source code.

- **Volumes** store provenance information that points to the underlying imagery data. This system is flexible to datastore, with extra functionality designed for BossDB users. In addition to dataset information, volumes include bounding box extents.
- **Graphs** store network-structured data composed of vertices and edges. Vertices and edges may contain metadata such as node type or edge direction. A Graph also points to a list of Decision objects corresponding to proofreader assessments of the validity of this graph. All vertices in a graph include a timestamp key which indicates when that particular vertex was created.
- **Nodes** store information about individual points in 3D space. Like Graph vertices, Nodes may include arbitrary metadata. Nodes likewise point to a list of Decisions assessing the validity of that Node annotation. Each Node includes a timestamp that indicates when that particular point was created.
- **Questions** store an individual task assignment and its metadata, including the name of the assignee, the Volume in which the task will take place, and what type of annotation is requested by the task author. Questions also include telemetry corresponding to the start- and end-times of a question, loosely corresponding to when a user began and completed a task. Questions also include the time of assignment (when the task was created) and a priority. This priority can be used by the Task Queue service to promote important tasks to be spooled and completed by a user sooner.
- **Decisions** store information relevant to users’ judgments on the validity of Graphs or Nodes. Decisions include telemetry such as the duration of time that a user took to make a decision, as well as the decision itself (“yes,” “no,” “maybe”).
- **Metadata** is stored to capture details pertaining to individual user accounts, including information about how many tasks the user has been assigned and how many tasks the user has completed, and an initial user categorization by experience level.

Database client interface libraries to manipulate data in the proofreading database are available in TypeScript and Python 3. The Python client supports numpy and pandas data standards [23], [24].

### C. Web Tool Development

Client-side web tools were developed using lightweight web technologies, including React for UI and state management and Substrate for 3D biomedical imagery visualization [25]. These tools were selected because they facilitate the use of composable, reusable components, which enabled us to rapidly develop and iterate on multiple purpose-built tools detailed above.

### D. Purpose-built applications enable rapid and accurate proofreading

Because each of our tasks was designed to prevent attentional wandering, we had the freedom to develop task-specific proofreading applications with featuresets to accommodate both novice and expert users. These applications were developed for modern web browsers in order to avoid the common pitfall of operating-system specific applications (see Methods). These tools enabled annotation or proofreading of keypoint data or graph data, the results of which could be used either as standalone data or as seeds to manipulate dense segmentation data. We show snippets of previously collected Pinky100 data [4], [22] in our visualizations.

#### 1) Keypoint “node” annotation:

**Fig. 2.**
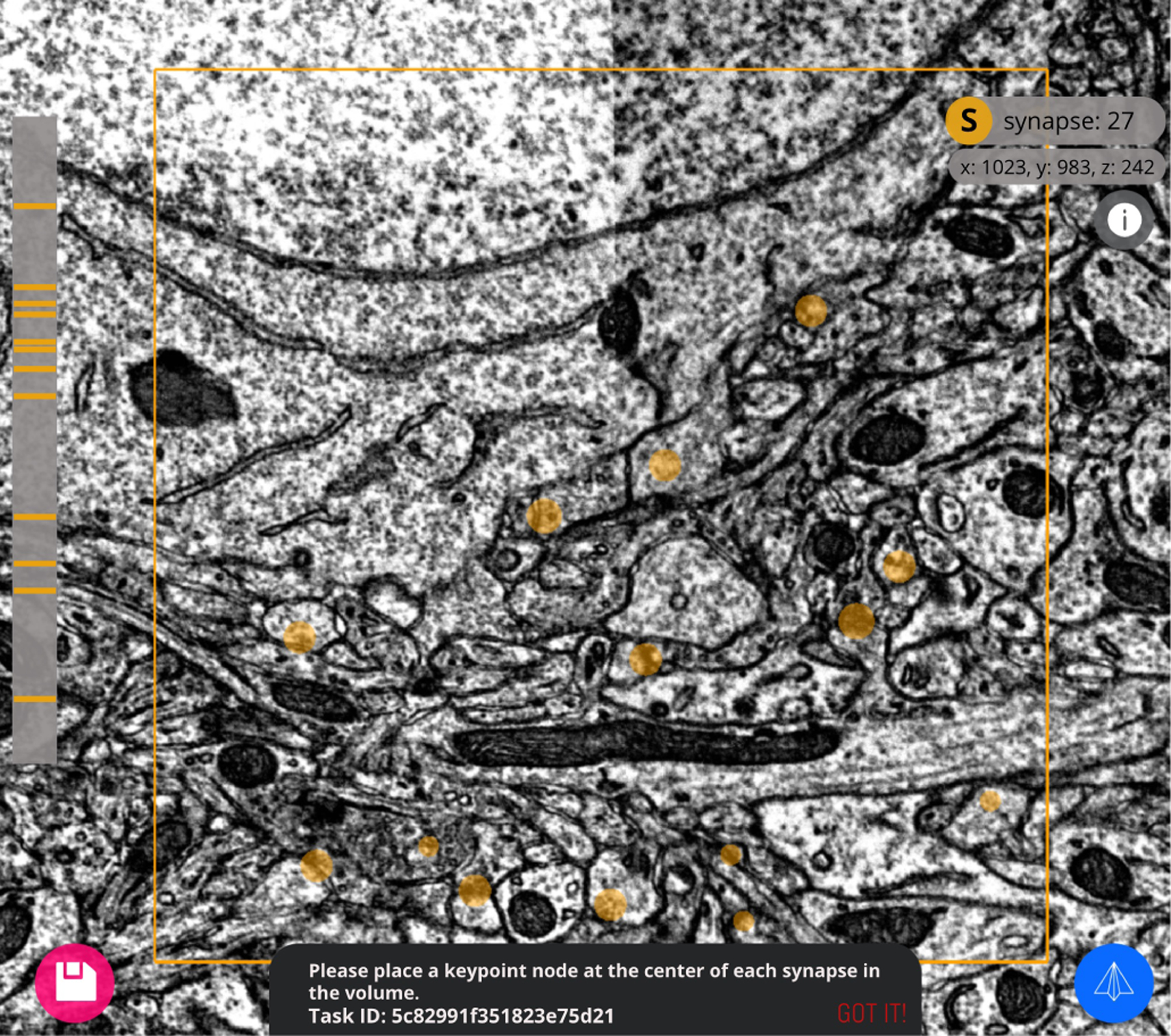
A synapse task in progress with the keypoint “node” annotation web application. In this example, the task instructs the user to annotate the center of each synapse in the 3D electron microscopy volume. A running count and index of synapse annotations are accessible to the user.

This application prompts users to drop individual keypoints in a 3D volume of known size. A set of “draft” keypoints can be provided by the task author for addition or removal by the user. Keypoints can represent anything, including high-resolution imagery annotation of synapses, low-resolution imagery annotation of cell bodies, artifact detection, or any other entity that can be represented by a single coordinate in 3D space (Figure 2).

#### 2) Keypoint “node” forced-choice proofreading:

**Fig. 3.**
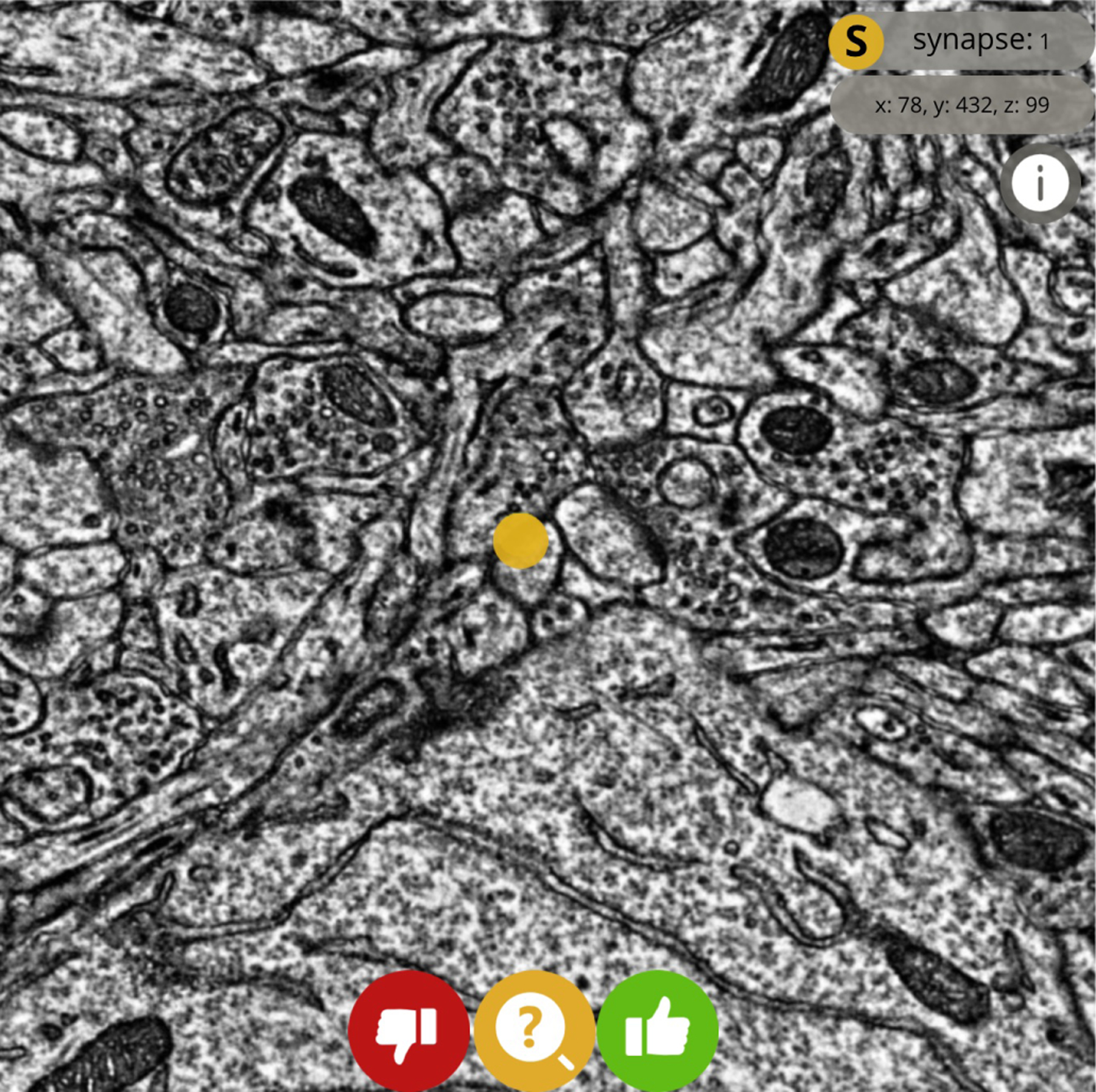
A forced-choice synapse proofreading task in progress. In this example, the task loads the image volume at the center of the annotated synapse of interest. The user can interact with the “yes,” “no,” and “maybe” buttons, or use keyboard shortcuts to complete the verification task.

The user is prompted to answer whether a single keypoint is a valid annotation or not. This application in particular enables extremely high-throughput proofreading. For example, when responding to synapse forced-choice proofreading, most users are able to answer within ten seconds. The user must respond with either a “yes” or “no” response; no proofreading activity is permitted. The task author may optionally also allow a third “maybe” option which users may select if they are not sure of the correct answer (Figure 3).

#### 3) Skeleton “graph” annotation:

The user is prompted to begin at a given starting point and trace from the starting point through the full extent of a single neuron, while remaining inside the task volume. All additional encountered synapses should be annotated, including the polarity of the encountered synapse (whether it performs as a presynaptic or postsynaptic actor in the traced neuron).

**Fig. 4.**
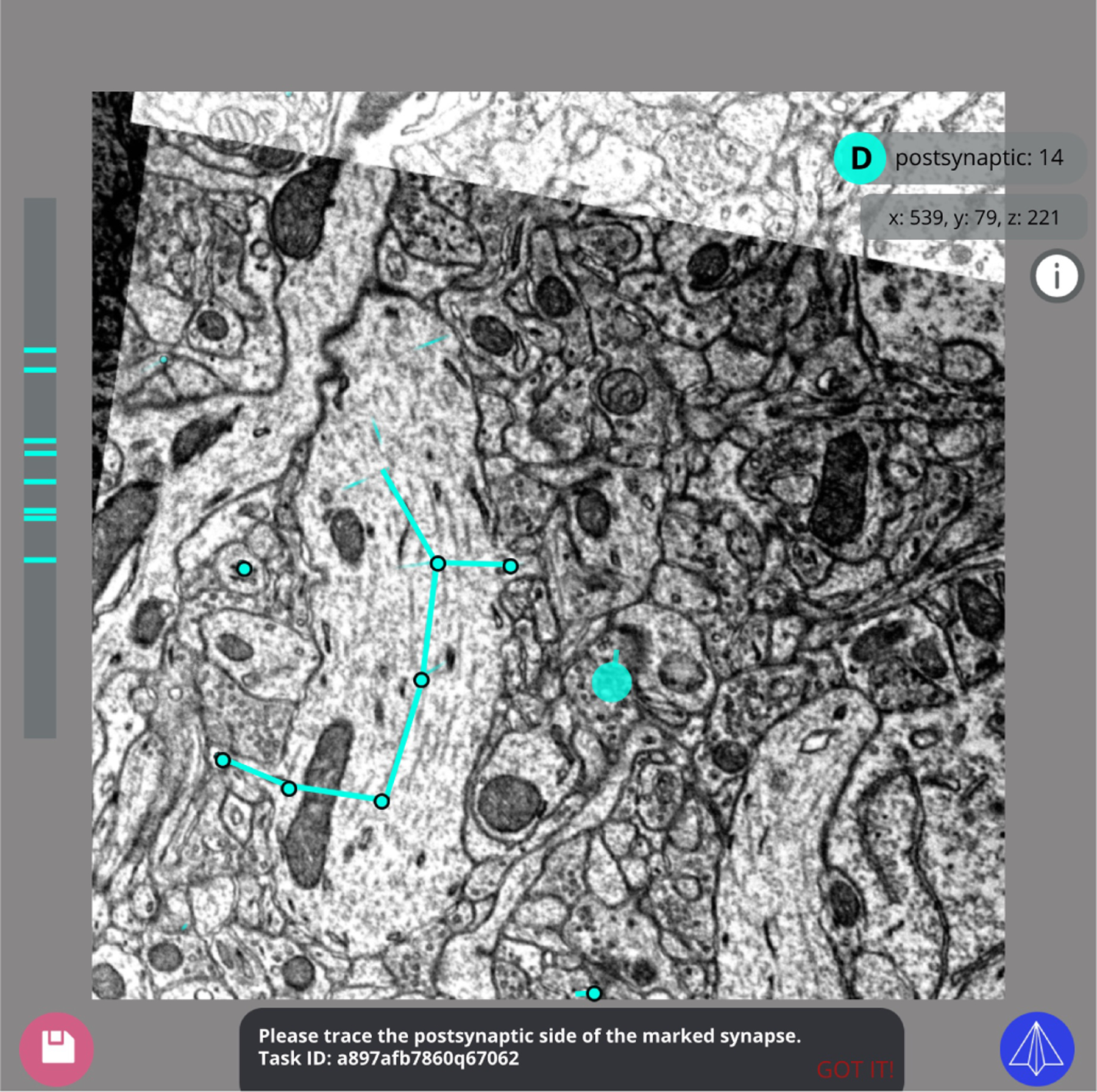
A tracing task in progress with the Skeleton “graph” annotation web application. In this example, the task instructs the user to trace the postsynaptic side of the marked synapse. The user can place intermediate “breadcrumb” nodes while navigating the 3D volume to maintain the structure of the neuron while annotating keypoints at each synaptic connection. A running count and locations of postsynaptic synapses annotated is accessible to the user.

This task may be repurposed to annotate any branching structure in 3D, including partially obscured splitting/merging entities in a 2D video. This makes it a valuable tool for behavioral studies in addition to 3D connectome reconstructions (Figure 4).

#### 4) Skeleton “graph” forced-choice proofreading:

**Fig. 5.**
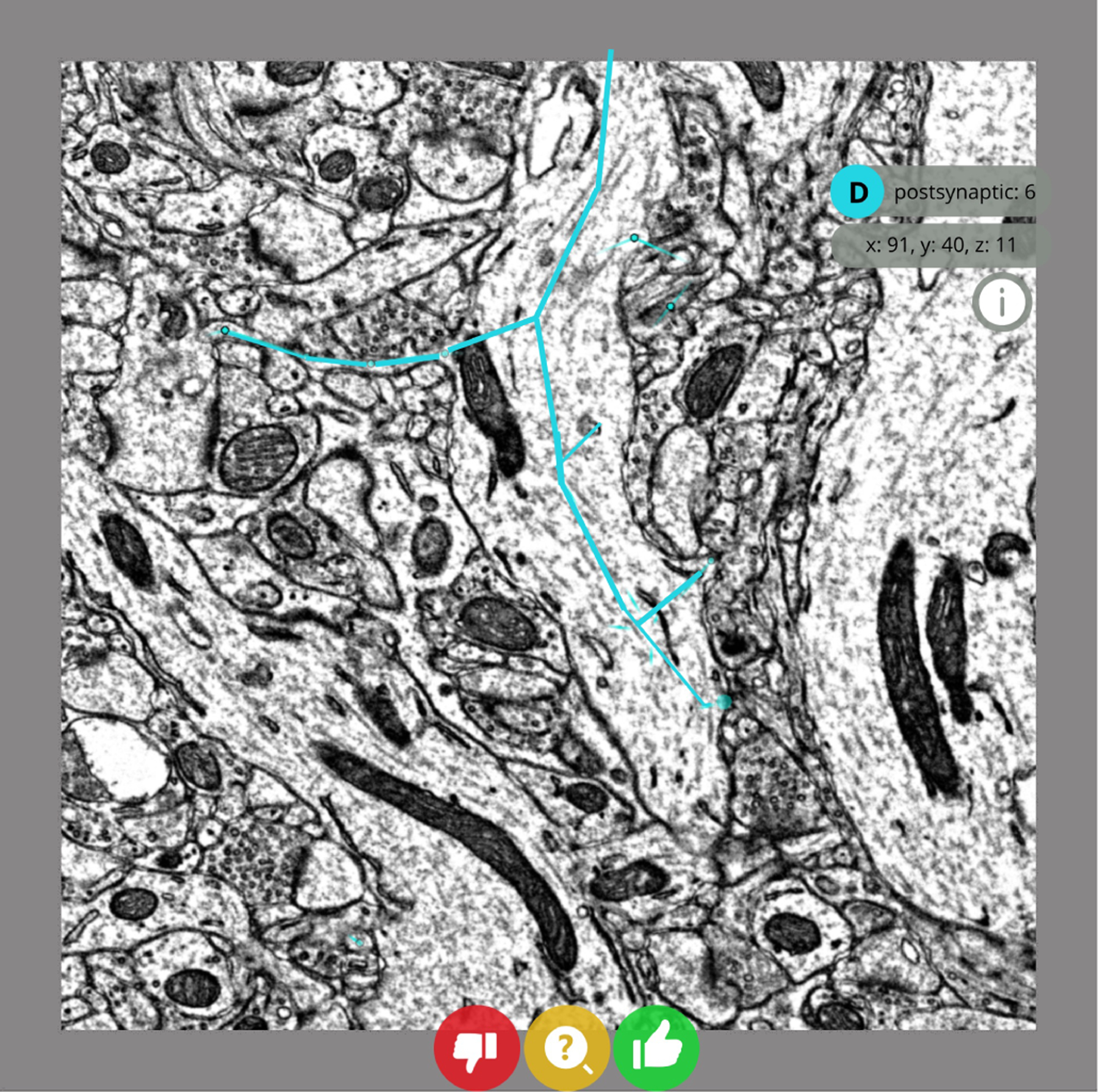
A forced-choice Skeleton “graph” proofreading task in progress. In this example, the task loads the image volume at the center of an annotated graph of interest. The user can interact with the “yes”, “no”, “maybe” buttons or use keyboard shortcuts to complete the verification task.

The application prompts a user to review a skeleton produced in the Graph Annotation tool for validity. The task author can allow the user to edit the skeleton in order to correct it or to simply evaluate it and provide a pass/fail response. Like the keypoint forced-choice tool, the task author may also optionally permit a third, “I Don’t Know / Maybe” option [26], [27] (Figure 5).

### E. Results Visualization

In order to provide non-expert users with valuable feedback, we developed a set of visualization tools to complement the proofreading applications. Users can proactively submit the ID of a completed task in order to see their response alongside the expert annotation or the annotation of their non-expert peers.

In addition to a 3D data visualization platform, we also provided annotators with a leaderboard, listing users in order according to a variety of metrics. These paired capabilities improved amateur annotator performance and encouraged friendly gamified competition among application users [28].

## III. Demonstration

Our proofreading workflow, dubbed CONFIRMS (Connectome Optimization of Networks For Informing Reconstructions and Motivating Science), was designed to address three major shortcomings of the current nanoscale connectomics process. First, we needed a way to accommodate varying levels of user expertise in a principled and data-driven way. Second, we needed an efficient and flexible pipeline for rapid connectome validation. Finally, we needed a system that was independent of the size of the dataset, and which could work for a gigabyte-scale image, a multi-petabyte scale image, and future exascale full-brain connectome datasets. During development, we iteratively deployed our tools, measured performance and throughput, and collected feedback from novice and expert users.

### A. Sampling

We devised several different strategies for sampling from large neuroimaging data volumes to be able to efficiently characterize data quality; we often use random sampling methods, but also developed the capabilities to sample by underlying image characteristics or biological properties (e.g., layers, synapse density). A typical proofreading problem will begin by calculating available resources and estimating the number of boxes to sample for both synapse and neuron fragment tasks. The relevant parameters are passed to the datastore (e.g., BossDB [18]) and the data is loaded into the proofreader applications on demand.

### B. Task Design

It is important that a proofreading system imposes minimal additional technical requirements on a neuroscience research program. For that reason, we rejected the possibility of time-consuming tasks such as whole neuron reconstruction (all tasks must take a novice user under 30 minutes, in order to avoid attentional errors) and we avoided systems that permit wandering from the spatial region of interest. This atomicity constrained our task design considerably, but also enabled us to support far wider spatial coverage in our tasks than would have been possible with conventional, “full-neuron” tracing tasks. Furthermore, we intend to enable tasks beyond neuron-tracing, where complete coverage of a single entity is either unnecessary (e.g., lung nodule identification in radiology) or impossible (e.g., tracing a region of space telescope data with non-uniform imaging resolution or spectrum).

When designing our task queue, we required that each task could be run statelessly and entirely independently of others (one task does not interfere with later tasks) and that tasks could be assigned directly to a user of a given experience level. Finally, in order to meet the atomic, short-duration task requirements set forth in Task Design, we developed several simple and user-friendly web applications to meet our specific data proofreading and annotation needs.

### C. User Types

There are a limited number of electron microscopy experts available to annotate at any time. In contrast, there is a large population of novice annotators with interest and investment in EM connectomics [28], [29]. While novice users may make more errors than an expert, it is possible in many cases to fuse multiple novice users’ annotations in order to reach expert-level accuracy, or to escalate particular points of disagreement to an expert. Furthermore, through feedback and training, a proofreading program such as this can rapidly improve user accuracy, reducing the number of non-expert annotations required to meet expert-annotation quality. We have used our tools with expert users (those with more than five full-time years of annotation experience), novice users (those starting with no previous EM annotation experience), and experienced users (those users that, through this pipeline, approached expert annotation levels). Feedback, financial compensation, and gamification are used to maintain user interest and attention. The atomic nature of these tasks allows for research teams to rapidly recruit and train proofreaders more efficiently than traditional workflows.

### D. Scoring and Evaluation

#### 1) Decision Fusion:

**Fig. 6.**
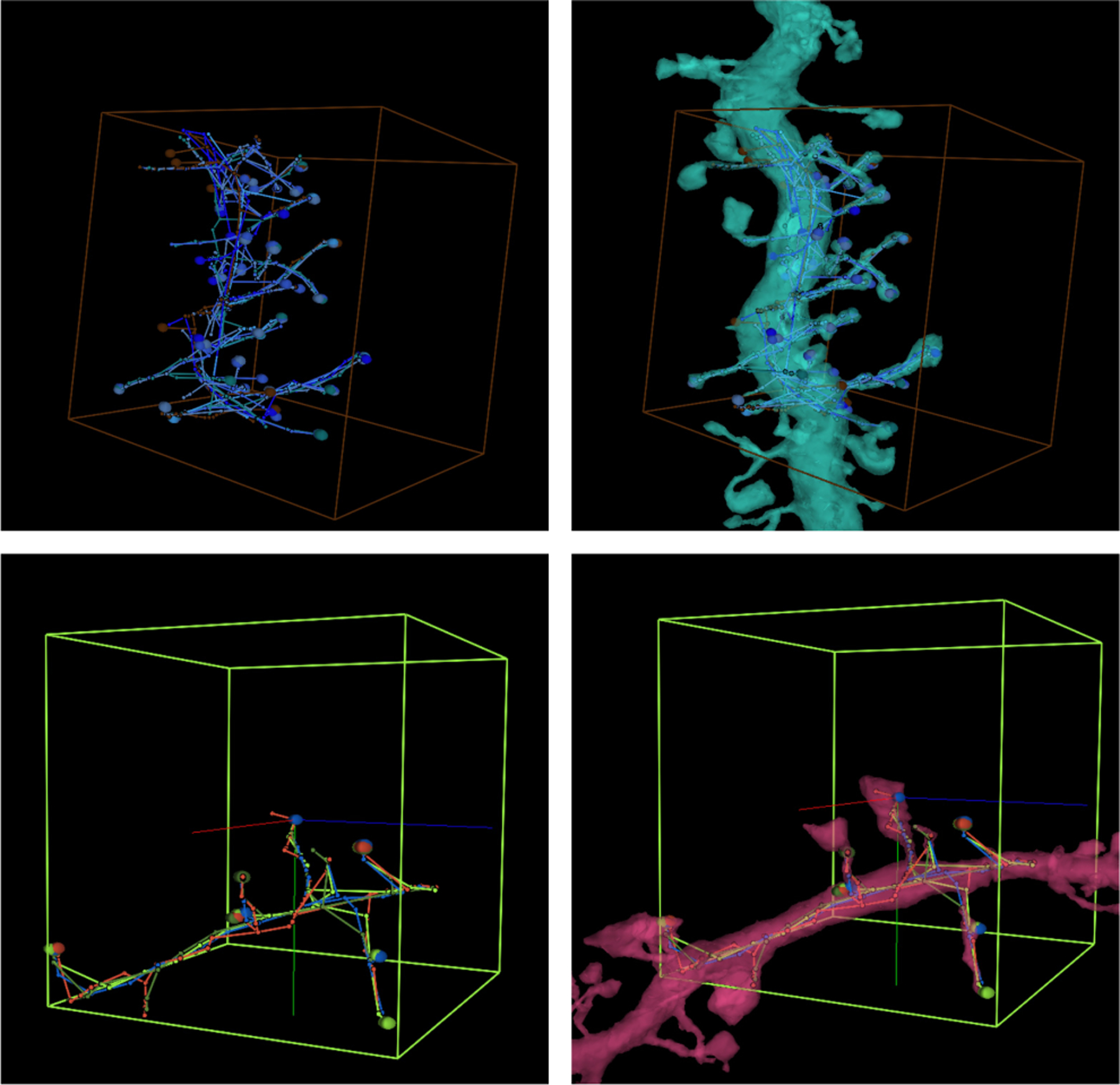
Fused novice Skeleton “graph” annotations with 3D meshed segmentation, illustrating the product of our visualizations with various overlays and also some of our visualization tools.

Because each task scheduled in our queue is small in spatial extent, an important stage in our connectome proofreading process is performing a task agreement step. Though our choice to make atomic, standalone tasks enabled high-throughput coverage of very large volumes in 3D space, it introduced two new challenges: fusion across multiple individual annotators for the same spatial extents, and fusion across multiple adjacent spatial extents (Figure 6). We combined multiple non-experts to approximate expert-level annotation quality (which needs to be validated empirically for a given annotator population, especially on difficult, less common morphologies). All adjacent annotation task volumes were designed to overlap by a sufficient margin to enable fusion across volume boundaries.

#### 2) Evaluation Workflows:

**Fig. 7.**
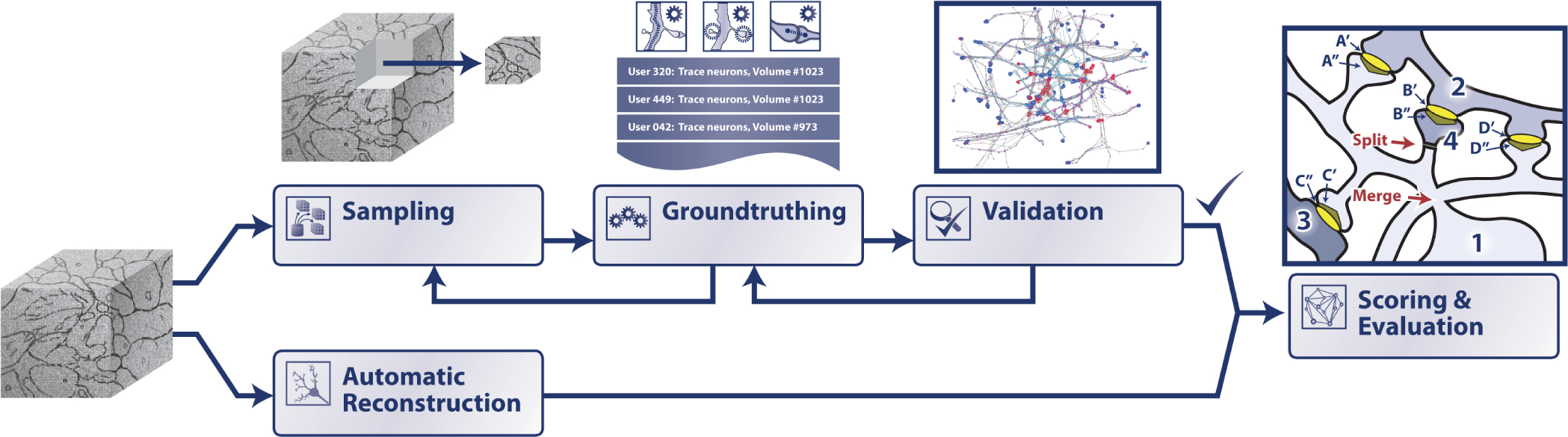
Overall CONFIRMS ecosystem connecting our proofreading and analysis tools into an evaluation workflow to assess connectome quality for downstream inference, based on an unseen (or “black box”) processing pipeline.

To further explain our workflows, we briefly describe our synapse and neuron scoring processes. In both cases, data-science and visualization-based validation (e.g., [25], [30]) were performed to help ensure an accurate evaluation (Figure 7). These can include larger-scale graph-based analyses, as well as sampling large processes for visualization and qualitative assessment to put our atomic tasks into context and provide quality assurance.

Connectomics pipelines have previously leveraged crowd-sourced citizen-science from the lay population [28], [31], but it was important here to not only allow multiple users to annotate a dataset but to weight their responses differently based upon the difficulty of a task, the complexity of a region of tissue, or the importance of a local region of interest. Our platform consists of short, simple stateless tasks that can be performed by a novice proofreader in under 30 minutes; a task queue and agreement system in order to convert these responses to connectivity measures with associated confidence measures; and a “render” step that converted these consolidated annotations to amended segmentation or other connectome byproducts, such as a synapse edgelist.

#### 3) Synapse Analysis:

We begin with randomly sampled synapse volumes of size 5×5×5 *μ*m (uniform random sampling) from the data volume (with edge padding). Annotators place keypoints on each synapse in subvolume; volumes require either one expert or 5 intermediate annotators with decision fusion (minimum agreement of 3). We filter out degenerate synapse volumes where synapse count was less than 5 or volumes that are heavily masked by imaging artifacts. We use the distance-based Hungarian-Munkres matching algorithm [32] (correcting for anisotropy) to match paired sets of our labeled synapses, treating one of the sets as ground truth. Each false positive (FP) and false negative (FN) result was checked by an expert and scores are adjusted accordingly. Ambiguous decisions are removed. Finally, we filter out duplicate synapses from keypoint sets and finalize the result into a discrete output. Precision, recall, and f1 values are used as primary metrics; in a typical task, we hope to see the variance of these metrics between volumes decrease as the number of sampled volumes increases.

#### 4) Neurons:

For neuron assessment, we randomly sample 1×1×1 *μ*m volumes (uniform random sampling) from the entire volume (excluding edges) to identify a series of starting seed points. An expert annotator places keypoints on every synapse in the sub-volume. We randomly sample a starting synapse as a starting location for axon and dendrite tracing (Axon volumes of size 12×12×12 *μ*m; Dendrite volumes of size 7×7×7 *μ*m with padding). Annotators start in the center of each volume and trace out neurites in both the pre- and post-synaptic directions. Volumes require either one expert or a minimum of five annotators per volume (fused with a minimum agreement of three). We again filter out degenerate volumes and compute NRI [9] for ground truth versus performer traces (although other metrics may be used instead). Each false positive (FP) and false negative (FN) edge is checked by experts and intermediate annotators and scores are adjusted. Ambiguous results are removed and results hardened to produce final scores.

### E. Usage Statistics

Over the lifetime of our applications, we have extensive user testing from approximately 60 users. In the last three years we have completed over 90,000 tasks (3,565 synapse volumes, 19,336 neurite traces, 46,758 forced-choice synapse tasks, and 22,282 forced-choice neurite tasks). A total of 244,663 synapses and 19,341 neurite graphs have been tagged. Workflows were run for targeted evaluations and so most of these annotations were collected over short periods.

## IV. Discussion

Similar to other big data research areas, the field of connectomics benefits enormously from the contributions of expert and non-expert users alike. Combining inputs from these heterogeneous users is a challenging and evolving problem space. In this work, we presented our solution for connectomics analysis tools and an associated workflow called CONFIRMS.

We identified a novel, rapid paradigm to apply modern data science methods to evaluate a graph reconstruction. In contrast to many efforts that optimize for high-quality morphological reconstructions or proofread large-scale segmentations, our primary usecase is to evaluate the quality of an underlying reconstruction through creating rapid annotations on small subvolumes of image data. Our workflows support both annotation and forced-choice decisions, allowing task queues to be focused on various tasks, including validating split and merge hypotheses. We leveraged many concepts from data science, such as clustering and combining decisions for a consensus estimate, and iteratively scheduling tasks to effectively leverage existing resources. We experimentally found that we could manage a team of dozens of heterogeneous proofreaders to rapidly characterize performance across several black box datasets segmented from an unseen source.

The tools developed as part of the CONFIRMS suite have user metrics and logging built in, in order to enable human factors research and user analysis. Future avenues for testing may include the efficacy and throughput of various workflows, sizing boxes to ensure that limited window views provide enough context to resolve challenging decisions, and more robust studies of interannotator agreement. We also have opportunities to study variability in annotator quality over time and on tasks of various complexity, as well as post-annotation decision metrics to eliminate attentional or intentionally poor annotations. From an infrastructure perspective, we have efficient tools to support scheduling and analysis, but the workflow requires a human in the loop to manage scheduling, select annotation boxes, and adjust task queues. Future work can automate many of these tasks for increased throughput and efficiency. When relying on non-expert annotators, additional analysis is required for each setting to ensure that data fusion truly approximates expert level scores and that systemic bias is not introduced. Additionally, when considering small subvolume sizes, the large-scale context must be considered to provide additional validation; we use visualization of entire neuron meshes and network-based measures as two quality checks.

This workflow represents a new way to approach the large volumes of connectomics data being produced today and to characterize datasets more quickly for computational neuroscience inference, medical understanding, and artificial intelligence. We have released the tool and analysis source-code at https://github.com/aplbrain/ to promote adoption and adaptation by the community.
